# Plant Cyclophilins: Multifaceted Proteins With Versatile Roles

**DOI:** 10.3389/fpls.2020.585212

**Published:** 2020-10-22

**Authors:** Harpreet Singh, Kirandeep Kaur, Mangaljeet Singh, Gundeep Kaur, Prabhjeet Singh

**Affiliations:** ^1^Department of Biotechnology, Guru Nanak Dev University, Amritsar, India; ^2^Department of Bioinformatics, Hans Raj Mahila Maha Vidyalaya, Jalandhar, India; ^3^William Harvey Heart Centre, Queen Mary University of London, London, United Kingdom

**Keywords:** cyclophilin, FKBP, hormones, immunophilins, peptidyl-prolyl *cis*-*trans* isomerase, stress

## Abstract

Cyclophilins constitute a family of ubiquitous proteins that bind cyclosporin A (CsA), an immunosuppressant drug. Several of these proteins possess peptidyl-prolyl *cis-trans* isomerase (PPIase) activity that catalyzes the *cis-trans* isomerization of the peptide bond preceding a proline residue, essential for correct folding of the proteins. Compared to prokaryotes and other eukaryotes studied until now, the cyclophilin gene families in plants exhibit considerable expansion. With few exceptions, the role of the majority of these proteins in plants is still a matter of conjecture. However, recent studies suggest that cyclophilins are highly versatile proteins with multiple functionalities, and regulate a plethora of growth and development processes in plants, ranging from hormone signaling to the stress response. The present review discusses the implications of cyclophilins in different facets of cellular processes, particularly in the context of plants, and provides a glimpse into the molecular mechanisms by which these proteins fine-tune the diverse physiological pathways.

## Introduction

A peptide bond in a folded protein can attain either *cis* or *trans* conformation, with the latter being favored due to geometrical and thermodynamic parameters (Ramachandran and Sasisekharan, 1968). However, the peptide bond preceding a proline (Pro) residue tends to adopt the *cis* configuration since its cyclic five-membered ring imposes rigid constraints on rotation about the N-C^α^ bond (Schulz and Schirmer, 2013). Hence, about 10–15% of peptidyl-prolyl bonds tend to adopt the *cis* conformation (Brandts et al., 1975). The presence of *cis*-proline peptide bonds has many structural implications as these tend to introduce bends in a protein and decrease stability. Therefore, *cis* to *trans* isomerization of peptide bonds, a rate-limiting process, is essential for the proper folding of proteins. Peptidyl-prolyl *cis-trans* isomerases (PPIases) are the only enzymes known that can catalyze *cis-trans* transition (Fischer et al., 1989). Unlike chaperones which require energy, the PPIases are typical enzymes that follow the Michaelis-Menten kinetics (Schmid et al., 1993; Fanghänel and Fischer, 2004).

The PPIases belong to three major classes of proteins *viz*., cyclophilins, FK506-binding proteins or FKBPs, and parvulins. While cyclophilins bind cyclosporin A (CsA), FKBPs and parvulins show interaction with FK506 (tacrolimus)/rapamycin (sirolimus) and juglone (5-hydroxy-1, 4-naphthoquinone), respectively. CsA and FK506 and its structural analog, rapamycin, are immunosuppressive drugs that are used for preventing graft rejection after allogeneic transplants (Göthel and Marahiel, 1999). These drugs block T-cell activation by interfering with the signal transduction pathways (Schreiber, 1991). The target of CsA was first detected in the bovine thymus as an 18 kDa protein, while the receptor for FK506 was identified as a protein of 12 kDa which was later also shown to bind to rapamycin (Handschumacher et al., 1984; Harding et al., 1989; Siekierka et al., 1989). The parvulins (Latin: parvulus, very small) were first identified in *E. coli* as a protein of 92 amino acid residues (Rahfeld et al., 1994). The PPIase activity of these proteins is sensitive only to juglone and is not affected by either CsA or FK506. Though cyclophilins and FKBPs are collectively referred to as immunophilins (Schreiber, 1991), members of these families show characteristics and conserved sequence features that differ between the two classes (He et al., 2004). Two new classes of PPIases *viz*., FCBPs (FK506 and CsA-binding proteins) that contain both cyclophilin and FKBP domains (Adams et al., 2005), and Protein Phosphatase 2A Phosphatase Activator (PTPA; Jordens et al., 2006) have also been discovered. While the FCBPs have not been reported in plants (Geisler and Bailly, 2007; Barik, 2018), the PTPA orthologs, though encoded by the plant genomes, have not been characterized yet for their PPIase activity (Chen et al., 2014).

Cyclophilins are ubiquitous proteins and are present in a wide range of organisms including viruses, bacteria, fungi, mammals and plants (Galat, 2003; Thai et al., 2008). Besides PPIase activity, a few members of this family also demonstrate chaperone activity, implying their multifaceted properties (Rinfret et al., 1994; Mayr et al., 2000; Marín-Menéndez et al., 2012). Recent advances in genome and transcriptome sequencing have revealed that relative to other organisms, the cyclophilin gene families show dramatic expansion in plants. The smallest and largest cyclophilin families with 29 and 94 genes have been reported in *Oryza sativa* and *Brassica napus*, respectively (Table 1). These proteins exhibit intra- and inter-specific differences in size (5.7 – 358.22 kDa) and pI values (4.4 – 12.6) (Table 1), suggesting divergence in their roles (Galat, 2004; Pemberton and Kay, 2005; Singh et al., 2019). Although inter- and intra-specific diversity of cyclophilins in plants indicates that these proteins may be performing distinct cellular functions (Table 2), with few of the roles being species-specific, the physiological significance of the majority of these proteins in plants is still a matter of conjecture. In the present article, we have attempted to summarize the different structural and functional aspects of cyclophilins in plants and their likely implications in different facets of growth and development.

**TABLE 1:** Genome-wide analysis of cyclophilins in different organisms.

Organism	Genes	Proteins	AAs	MW (kDa)	pI	SD	MD	Localization	References
**Plants**									
*Arabidopsis thaliana*	31	48	151-837	15.9-94.6	4.5-12.60	40	08	Ch (4), Ch/P (7), Ch/ER (2), Ch/M (3), Cy (14), Cy/ER (1), CS (1), E/ER (3), N (10), V/ER (2), V/M (1)	Kumari et al., 2015
*Brassica napus*	94	91	49-1268	5.7-146.1	4.4-11.80	79	12	Ch (14), Cy (50), M (7), N (13), S (7)	Hanhart et al., 2017
*Glycine max*	62	62	114-850	12.4-96.20	4.97-11.74	52	10	Ch (13), Cy (21), M (5), N (10), SP (13)	Mainali et al., 2014
*Gossypium barbadense*	75	75	78-1256	8.50-142.5	4.5-11.3	61	14	Ch (7), Ch/Cy (1), Ch/E (1),Cy (37), Cy/N (3), Cy/E (1), Cy/M (1), M (1), N (13), E (7), M/E (1), M/N (2)	Chen et al., 2019
*G. hirsutum*	78	78	112-828	12.0-92.89	4.9-11.50	60	18	Ch (10), Ch/E (3), Cy (36), Cy/Ch/M (1), Cy/N (1), E/Cy (2), M (3), N (10), E (6), M/Cy (1), M/N (5)	Chen et al., 2019
*G. arboretum*	40	40	149-795	15.65-89.80	4.9-11.50	32	08	Ch (5), Ch/Cy (1), Cy (19), Cy/Ch/M (1), Cy/M (1), M (2), N (4), PM (1), E (4), N/M (1), PM/N/Cy (1)	Chen et al., 2019
*G. raimondii*	38	38	164-801	18.03-90.62	4.9-11.50	29	09	Ch (4), Ch/E (1), Cy (16), Cy/Ch (3), E/Cy (1), E (2), E/N (1), M (1), N (4), M/N (2), N/Cyl (1), PM (1)	Chen et al., 2019
*Medicago truncatula*	33	33	125-895	NA	NA	21	12	Cy, Ch, CS, ER, G, M, N, PM, PS	Ge Q. et al., 2020
*Oryza* sativa	29	46	139-1089	16.2-124	4.5-11.50	38	8	Ch (6), Ch/P (3), Ch/ER (5), Cy (1l), Cy/M (2), Cy/ER (1), CS (2), N (8), V/ER (1), M/P (2), M (5)	Kumari et al., 2015
*Triticum aestivum*	83	85	160-823	17.2-92.08	4.76-11.53	58	27	Cy (28), Ch (22), M (09), N (19), N/ER (3), S (4)	Singh et al., 2019
**Animal**									
*Homo sapiens*	17	19	161-3224	18.0-358.22	5.3-10.70	12	07	C, ER, M, N, SS	Galat, 2004; Schiene-Fischer, 2015
**Protozoa**									
*Plasmodiophora brassicae*	11	11	NA	NA	NA	07	04	Cy (8), E (1), M (2)	Singh et al., 2018
**Fungi**									
*Leptosphaeria maculans*	12	12	165-663	16.8-74.1	5.01-9.46	08	04	Cy (8), M (1), N (3)	Singh et al., 2014
*Purpureocillium lilacinum*	10	11	162-627	17.4-70.20	5.8-9.50	06	04	Cy (6), ER (1), M (1), N (2)	Mo et al., 2019
*Phytophthora sojae*	20	20	166-630	18.3-69.80	NA	15	05	M (3), S (3)	Gan et al., 2009
*P. ramorum*	21	21	163-633	18.0-70.20	NA	16	05	M (2), S (3)	Gan et al., 2009
*P. infestans*	21	21	161-630	17.5-69.60	NA	16	05	M (2), S (3)	Gan et al., 2009
*Saccharomyces cerevisiae*	08	08	162-393	17.4-45.1	5.1-9.10	06	02	C (2), C/N (2), V (2), M (1), ER (1)	Arevalo-Rodriguez et al., 2004; Galat, 2004
*Schizosaccharomyces pombe*	09	09	155-610	16.8-69.0	5.5-9.20	05	04	C and ER	Galat, 2004
**Bacterium**									
*Escherichia coli*	02	02	164-190	21-22	5.0-9.70	02	00	C and P	Liu and Walsh, 1990; Hayano et al., 1991

*AAs, Amino acids; C, Cytoplasm; Ch, Chloroplast; CS, Cytoskeleton; Cy, Cytosol; E, Extracellular; ER, Endoplasmic reticulum; G, Golgi; L, Lumen; M, Mitochondria; MD, Multi domain; MW, Molecular weight; N, Nucleus; NA, Information not available; P, Periplasm; PM, Plasma membrane; pI, Isoelectric point; PS, Peroxisome; S, Secreted; SD, Single domain; SP, Signal peptide; SS, Spliceosome; V, Vacuole.*

**TABLE 2:** Cellular functions of cyclophilins in different organisms.

Organism	Gene	Protein	Localization	SD/MD	MW (kDa)	Proposed Functions	References
**Plants**							
*Arabidopsis thaliana*	*AtCYP18-3/ROC1*	AtCYP18-3/ROC1	Cytosol	SD	18.40	Plant pathogen interaction, brassinosteroid signaling	Coaker et al., 2006; Trupkin et al., 2012
	*AtCYP19-1/ROC3*	AtCYP19-1/ROC3	Cytosol	SD	18.50	Seed development, plant-pathogen interaction	Stangeland et al., 2005; Pogorelko et al., 2014
	*AtCYP19-2/ROC6*	AtCYP19-2/ROC6/AtCYP2	Cytosol	SD	18.50	Differentiation or development of foliar organs	Chou and Gasser, 1997; Saito et al., 1999b
	*AtCYP19-4/AtCYP5*	AtCYP5/CYP5	Secretory protein	SD	19.00	Regulation of embryogenesis	Grebe et al., 2000; Romano et al., 2004b
	*AtCYP20-1/ROC7*	AtCYP20-1/ROC7	Secretory protein	SD	19.60	Regulation of PP2A activity	Jackson and Söll, 1999; Romano et al., 2004b
	*AtCYP20-2*	AtCYP20-2	Thylakoid luminal protein	SD	20.00	Biogenesis of NDH complexes	Sirpiö et al., 2009
	*AtCYP20-3/ROC4*	AtCYP20-3/ROC4	Chloroplast stromal protein	SD	19.80	Modulates retrograde signaling, folding and assembly of SAT-1 enzyme, links redox and light signals to cysteine biosynthesis	Romano et al., 2004b; Dominguez-Solis et al., 2008; Kopriva, 2013
	*AtCYP38/CYP38*	AtCYP38/CYP38	Thylakoid luminal protein	MD	38.30	Assembly and maintenance of PS-II	Romano et al., 2004b; Fu et al., 2007; Sirpiö et al., 2009
	*AtCYP57*	AtCYP57	Cytosol	MD	57.10	Plant defense	Pogorelko et al., 2014
	*AtCYP59*	AtCYP59	Cytosol	MD	58.80	Regulates pre-mRNA processing	Gullerova et al., 2006
	*AtCYP65*	AtCYP65	Cytosol	MD	63.50	Molecular chaperone and prevents protein aggregation	Wiborg et al., 2008
	*AtCYP71/CYP71*	AtCYP71/CYP71	Cytosol	MD	70.70	Regulates gene expression and organogenesis	Li et al., 2007; Li and Luan, 2011; Romano et al., 2004b
	*AtCYP95*	AtCYP95	Nucleus	MD	94.60	Pre-mRNA splicing	Lorkoviæ et al., 2004
*Citrus sinensis*	*CsCYP*	CsCYP	Nucleus	SD	18.00	Interacting partner for RNA polymerase-II, key player in transcriptional cycle.	Domingues et al., 2012
*Lycopersicon esculentum*	*DGT*	LeCYP1	Cytosol	SD	17.90	Auxin signaling	Gasser et al., 1990; Ivanchenko et al., 2006
*Medicago truncatula*	*MsCYP20-3B*	MsCYP20-3B	Chloroplast	SD	20	Regulate axillary shoot development	Ge Q. et al., 2020
*Oryza sativa*	*OsCYP-2*	OsCYP2	Cytosol and nucleus	SD	18.30	Regulation of initiation of lateral roots	Wang et al., 2007; Kumari et al., 2009; Zheng et al., 2013
*Panax ginseng*	*PgCYP*	PgCYP	Cytosol	SD	18.70	Antifungal activity	Zhang et al., 2017
*Ricinus communis*	*RcCYP1*	RcCYP1	Cytosol	SD	29.00	Refolding of non-autonomous proteins	Gottschalk et al., 2008
*Spinach oleracea*	*TLP40*	TLP40	Thylakoid-lumen	MD	40.00	Regulation of activity of PS-II specific protein phosphatase	Fulgosi et al., 1998; Edvardsson et al., 2003
*Triticum aestivum*	*TaCYP20-2*	TaCYP20-2	Thylakoid lumen	SD	25.80	Regulates flowering	Zhang et al., 2013b
**Animal**							
*Homo sapiens*	*PPIA/CYPA*	CYPA	Cytosol	SD	18.00	Regulation of infectivity of HIV virions, cancer cell proliferation, chaperone	Braaten and Luban, 2001; Obchoei et al., 2009; Zhang et al., 2013a
	*PPIB/CYPB*	CYPB	ER	SD	22.00	Regulation of Hepatitis C virus replication, activation of IRF3	Galat, 2004; Watashi et al., 2005; Obata et al., 2005
	*PPIC/CYPC*	CYPC	Cytoplasm/ER	SD	22.70	Activation of macrophages	Galat, 2004; Yamaguchi et al., 2011
	*PPIF/CYPD*	CYPD	Mitochondria	MD	40.70	Protection from cell death, regulator of mitochondria permeability transition pore	Lin and Lechleiter, 2002; Galat, 2004; Schubert and Grimm, 2004; Elrod et al., 2010
	*PPIE/hCYP33*	hCYP33	Nucleus	MD	33.40	mRNA processing, transcription regulation	Wang et al., 2008
	*NKTR*	*NKTR* (NK tumor recognition protein)	Cell membrane	MD	165.60	NK tumor recognition complex molecule	Anderson et al., 1993
**Yeast**							
*Saccharomyces cerevisiae*	*CPR1/CYP1*	CPR1	Cytosol and nucleus	SD	17.00	CsA receptor, regulation of meiosis	Sykes et al., 1993; Breuder et al., 1994; Arévalo-Rodríguez and Heitman, 2005
	*CPR2/CYP2*	CPR2	ER	SD	20.50	Enhances cell survival in response to heat shock	Sykes et al., 1993; Dolinski et al., 1997
	*CPR3*	CPR3	Mitochondria	SD	20.00	Lactate metabolism, protein folding	Davis et al., 1992; Matouschek et al., 1995
	*CPR6*	CPR6	Cytosol	SD	45.00	Hsp90 binding, interaction with Ura2 (critical protein for pyrimidine biosynthesis)	Zuehlke et al., 2013
	*CPR7*	CPR7	Cytosol	MD	45.00	Hsp90 interaction, heat shock response regulator,	Duina et al., 1998
**Bacterium**							
*Escherichia coli*	*PpiA*	PPIA	Periplasm	SD	18.13	Folding of secreted proteins	Lazar and Kolter, 1996
	*PpiB*	PPIB	Cytosol	SD	18.18	Unknown	Hayano et al., 1991

*The nomenclature of genes and proteins is according to the respective publications. ABA, Abscisic acid; CsA, Cyclosporin A; ER, Endoplasmic reticulum; GA, Gibberellic acid; IRF3, interferon regulatory factor 3; MD, Multi domain; MW, Molecular weight; NDH, NADH dehydrogenase; NK, Natural killer; PP2A, Protein phosphatase 2A; PS, Photosystem; SAT-1, Serine acetyltransferase; SD, Single domain; Ura2, uracil-aspartate 2.*

### Structural Analyses of Cyclophilin Genes and Proteins in Plants

Genome-wide analyses revealed that the distribution of cyclophilin genes on different chromosomes in plants is uneven (Table 3). The cyclophilin genes in allopolyploids such as *B. napus* and wheat occur in pairs, with each member originating from one progenitor chromosomal set. These pairs are highly identical and share localization patterns (Hanhart et al., 2017; Singh et al., 2019). Structural analysis of cyclophilin genes in plants has been carried out for soybean, cotton, wheat and *Medicago truncatula* (Mainali et al., 2014; Chen et al., 2019; Singh et al., 2019; Ge L. et al., 2020). These studies revealed considerable variability in the distribution and size of introns in the open reading frames (ORFs) and untranslated regions (UTRs) as compared to other organisms (Table 4). The cyclophilin genes with the highest number of introns include cotton (20 in *GbCYP142;* Chen et al., 2019), wheat (13 each in *TaCYP64-1-7A, TaCYP64-2-7B*, and *TaCYP64-3-7D*; Singh et al., 2019) and soybean (13 each in *GmCYP56* and *GmCYP59*; Mainali et al., 2014). The largest intron (28618 bp) was observed in *TaCYP26-5-2B*, while the smallest (39 bp) was noticed in *GmCYP5* (Table 4). Information about variations in the structure of cyclophilin genes in rice, *Arabidopsis* and *Brassica*, which is lacking, may provide further insights into the evolution of these families in plants. Loss or gain of introns, an important aspect of structural variation, is vital for gene evolution (Roy and Gilbert, 2006). The intron size may be correlated with the genome size and longer introns have been proposed to confer a selective advantage by improving the recombination, and also by counterbalancing the mutational bias towards deletions (Carvalho and Clark, 1999; McLysaght et al., 2000). Thus, the variability in introns in plant cyclophilins may have important implications in their functionalization which needs to be explored further. Since 5′ and 3′ UTRs are structurally important and regulate the expression of eukaryotic genes (Wilkie et al., 2003), differences in these regions may likely enable differential regulation of plant cyclophilins, leading to divergence in their physiological roles.

**TABLE 3:** Chromosomal distribution of cyclophilin genes in plants.

Organism	Chromosome	Cyclophilin Genes	No. of Tandem/Segmental Duplicated Gene Pairs	References
*Arabidopsis thaliana*	Chr1	05	NA	Kumari et al., 2015
	Chr2	07		
	Chr3	09		
	Chr4	06		
	Chr5	04		
*Brassica napus*	A01	11	NA	Hanhart et al., 2017
	A02	03		
	A03	03		
	A04	03		
	A05	05		
	A06	03		
	A07	01		
	A08	05		
	A09	06		
	A10	02		
	C01	05		
	C02	03		
	C03	08		
	C04	10		
	C05	04		
	C06	01		
	C07	03		
	C08	07		
	C09	03		
*Glycine max*	Ch1	04	16 (tandem)	Mainali et al., 2014
	Ch2	03		
	Ch3	04		
	Ch4	03		
	Ch5	02		
	Ch6	04		
	Ch7	02		
	Ch8	00		
	Ch9	03		
	Ch10	03		
	Ch11	06		
	Ch12	04		
	Ch13	03		
	Ch14	01		
	Ch15	04		
	Ch16	00		
	Ch17	04		
	Ch18	03		
	Ch19	06		
	Ch20	03		
*Gossypium barbadense*	A01	NA	02 (tandem) 39 (segmental)	[Bibr B37][Bibr B37][Bibr B37][Bibr B37]
	A02	NA		
	A03	NA		
	A04	NA		
	A05	NA		
	A06	NA		
	A07	NA		
	A08	NA		
	A09	NA		
	A10	NA		
	A11	NA		
	A12	NA		
	A13	NA		
	D01	NA		
	D0	NA		
	D03	NA		
	D04	NA		
	D05	NA		
	D06	NA		
	D07	NA		
	D08	NA		
	D09	NA		
	D10	NA		
	D11	NA		
	D12	NA		
	D13	NA		
*G. hirsutum*	AD1-D01	04	03 (tandem)	
	AD1-D02	03		
	AD1-D03	02		
	AD1-D04	03		
	AD1-D05	01		
	AD1-D06	02		
	AD1-D07	04		
	AD1-D08	05		
	AD1-D09	02		
	AD1-D10	03		
	AD1-D11	02		
	AD1-D12	03		
	AD1-D13	02		
*G. arboretum*	A2-chr1	05	02 (tandem)	
	A2-chr2	02		
	A2-chr3	05		
	A2-chr4	02		
	A2-chr5	03		
	A2-chr6	04		
	A2-chr7	06		
	A2-chr8	01		
	A2-chr9	02		
	A2-chr10	01		
	A2-chr11	02		
	A2-chr12	04		
	A2-chr13	03		
*G. raimondii*	D5-chr1	03	01 (tandem)	
	D5-chr2	02		
	D5-chr3	01		
	D5-chr4	01		
	D5-chr5	02		
	D5-chr6	03		
	D5-chr7	03		
	D5-chr8	06		
	D5-chr9	03		
	D5-chr10	03		
	D5-chr11	02		
	D5-chr12	03		
	D5-chr13	01		
*Medicago truncatula*	Chr1	05	07 (segmental)	Ge Q. et al., 2020
	Chr2	03		
	Chr3	05		
	Chr4	04		
	Chr5	04		
	Chr6	02		
	Chr7	05		
	Chr8	05		
*Oryza sativa*	Ch1	03	NA	Kumari et al., 2015
	Ch2	04		
	Ch3	03		
	Ch4	00		
	Ch5	01		
	Ch6	06		
	Ch7	03		
	Ch8	04		
	Ch9	02		
	Ch10	02		
	Ch11	01		
	Ch12	00		
*Triticum aestivum*	Chr1A	01	06 (tandem) 15 (segmental)	Singh et al., 2019
	Chr2A	02		
	Chr3A	04		
	Chr4A	05		
	Chr5A	03		
	Chr6A	06		
	Chr7A	09		
	Chr1B	01		
	Chr2B	03		
	Chr3B	05		
	Chr4B	04		
	Chr5B	02		
	Chr6B	06		
	Chr7B	08		
	Chr1D	01		
	Chr2D	03		
	Chr3D	05		
	Chr4D	04		
	Chr5D	02		
	Chr6D	04		
	Chr7D	09		

*NA, Information not available.*

**TABLE 4:** Variability in architecture of cyclophilin genes.

Organism	Gene	Number of Exons	Size Range of Introns	No. of Introns in			Cyclophilin genes lacking introns	References
				**ORF**	**5′UTR**	**3′UTR**		
**Plants**								
*Glycine max*	62	1-14	39 bp (*GmCYP5*); 9359 bp (*GmCYP56*)	0-13	0-1	0-5	*GmCYP1*(973 bp), *GmCYP2* (1224 bp), *GmCYP3* (854 bp), *GmCYP4* (775 bp), *GmCYP6* (373 bp), *GmCYP7* (1072 bp), *GmCYP11* (1062 bp)	Mainali et al., 2014
*Gossypium barbadense*	75	1-21	–	0-20	–	–	*GbCYP14-2*, *GbCYP16-1*, *GbCYP18-1*, *GbCYP18-2*, *GbCYP18-3*, *GbCYP18-4*, *GbCYP18-5*, *GbCYP18-6*, *GbCYP18-7*, *GbCYP18-8*, *GbCYP18-9*, *GbCYP24-1*	Chen et al., 2019
*G. hirsutum*	78	1-14	–	0-13	–	–	*GhCYP12*, *GhCYP18-2*, *GhCYP18-3*, *GhCYP18-4*, *GhCYP18-5*, *GhCYP18-6*, *GhCYP18-7*, *GhCYP18-8*, *GhCYP18-9*, *GhCYP18-11*, *GhCYP18-12*	
*G. arboreum*	40	1-15	–	0-14	–	–	*GaCYP15*, *GaCYP18-3*, *GaCYP18-4*, *GaCYP18-5*, *GaCYP18-6*, *GaCYP18-7*	
*G. raimondii*	38	1-14	–	0-13	–	–	*GrCYP18-2*, *GrCYP18-3*, *GrCYP18-4*, *GrCYP18-5*, *GrCYP18-6*, *GrCYP18-7*	
*Medicago truncatula*	33	1-14	–	0-13	–	–	*MtCYP19-1A*, *MtCYP19-1B*, *MtCYP19-3*, *MtCYP40B*	Ge Q. et al., 2020
*Triticum aestivum*	83	1-14	78 bp (*TaCYP41-2-7A* & *TaCYP41-3-7B*); 28618 bp (*TaCYP26-5-2B*)	0-13	0-1	0-1	*TaCYP17-4-6A* (504 bp), *TaCYP18-4-6A* (973 bp), *TaCYP18-4-6D* (969 bp), *TaCYP18-5-6B* (903 bp), *TaCYP18-6-4B* (540 bp), *TaCYP23-2-6B* (660 bp), *TaCYP24-1-6B* (660bp), *TaCYP26-1-3B* (771 bp), *TaCYP26-6-6A* (3785 bp), *TaCYP45-1-3A* (1218 bp), *TaCYP54-1-4A* (1437 bp)	Singh et al., 2019
**Fungi**								
*Purpureocillium lilacinum*	10	1-6	–	0-5	–	–	*-*	Mo et al., 2019
*Phytophthora sojae*	20	1-8	–	0-7	–	–	*Ps*, *Ps2*, *Ps4*, *Ps6*, *Ps7*, *Ps10*, *Ps13*, *Ps20*	Gan et al., 2009
*P. ramorum*	21	1-7	–	0-6	–	–	*Pr1*, *Pr4*, *Pr7*, *Pr10*, *Pr11*, *Pr13*, *Pr14*	Gan et al., 2009
*P. infestans*	21	1-6	–	0-5	–	–	*Pi1*, *Pi4*, *Pi6*, *Pi7*, *Pi10*, *Pi13*, *Pi14*, *Pi20*	Gan et al., 2009

*The nomenclature of genes used is according to the respective publications. ORF, Open reading frame; UTR, Untranslated region.*

**FIGURE 1:**
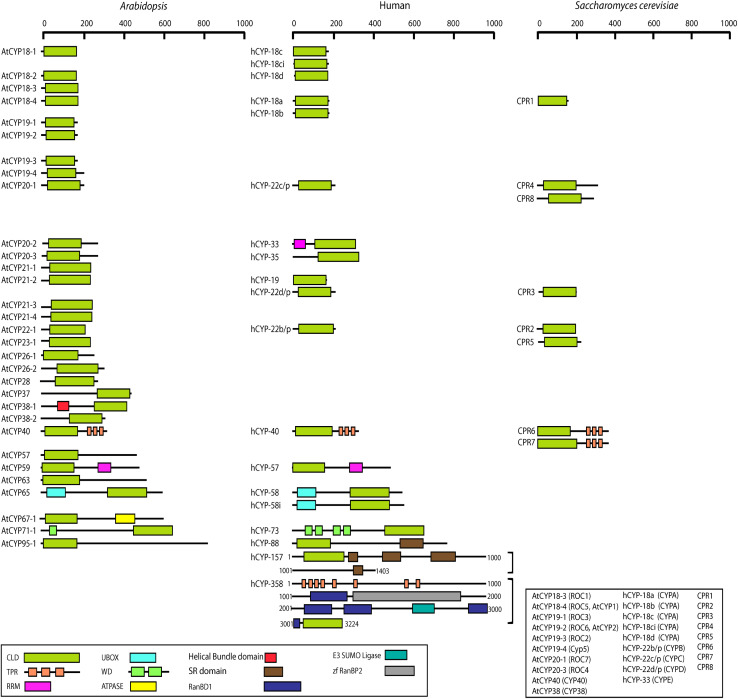
Comparative analysis of domain architecture of *Arabidopsis* cyclophilins with their orthologs in human and *Saccharomyces cerevisiae*. The amino acid residues that define the protein domains are designated according to Galat (2004), Wang and Heitman (2005), Kumari et al. (2015), and Schiene-Fischer (2015). For *Arabidopsis* cyclophilins that may have alternatively spliced forms, the domain architecture is shown for only a single variant. CLD, cyclophilin-like domain; RRM, RNA recognition motif; TPR, tetratricopeptide repeat; U-box, U box domain; WD40, WD40 repeat; RanBDl, Ran binding protein 1 domain; zf RanBP, Zn-finger, Ran-binding; SR, Serine arginine rich domain. The nomenclature and alternative protein names are given in the box. Scale bar represents the length of amino acid sequence.

The cyclophilins in plants and other organisms, though predominantly cytosolic, are also predicted to be localized in the chloroplast, nucleus, mitochondria, extracellular/secretory and plasma membrane (Table 1). The presence of cyclophilins in different organelles of plants signifies their specific and distinct roles in the cell (Tables 1, 2). Based on domain organization, the cyclophilins are classified as single- (SD) or multi-domain (MD) forms (Table 1). The SD cyclophilins possess the characteristic cyclophilin-like domain (CLD), while the MD cyclophilins also contain additional specific functional domains (Table 5). Analysis of CLD in the typical human cyclophilin, hCYPA (hCYP18-A/CYPA), demonstrated that the residues Arg55, Phe60, Met61, Glu63, Ala101, Phe113, Trp121, Leu122 and His126 are essential for PPIase activity (Zydowsky et al., 1992b; Ke et al., 1994; Zhao et al., 1997; Howard et al., 2003; Davis et al., 2010). Arg55, in particular, plays a critical role in PPIase functions, whereas Trp121, though not involved in *cis-trans* isomerization, is essential for CsA binding (Liu et al., 1991; Zydowsky et al., 1992b; Howard et al., 2003). Interestingly, in the plant MD cyclophilins, the TPR and WD40 repeats are observed more commonly compared to other domains (Table 5). The domains such as TPR, WD40, F-box, coiled-coil, etc., have been reported to facilitate protein-protein interactions in the cell (Lamb et al., 1995; Craig and Tyers, 1999; Van Nocker and Ludwig, 2003; Liu et al., 2006). Hence, the cyclophilins consisting of these motifs may be acting as platforms for assembling protein complexes or mediate transient interactions among other proteins, further indicating their functional versatility (Bandziulis et al., 1989; Van Nocker and Ludwig, 2003; Stirnimann et al., 2010; Earley and Poethig, 2011). Compared with yeast and human cyclophilins, the presence of various additional domains such as PsbQ-like, F-box, Helical bundle, ATPase and PAN_4 domain in the plant MD cyclophilins (Figure 1 and Table 5) signifies divergence of their roles that are yet to be explored completely (Dornan et al., 2003; Romano et al., 2004b; Mainali et al., 2014; Kumari et al., 2015; Hanhart et al., 2017; Chen et al., 2019; Singh et al., 2019).

**TABLE 5:** Comparative analysis of functional domains (other than cyclophilin-like domain) in the different multi-domain cyclophilins.

Domain	Role	*Arabidopsis thaliana*	*Brassica napus*	*Glycine max*	*Gossypium sp.*	*Medicago truncatula*	*Oryza sativa*	*Triticum aestivum*	*Homo sapiens*	References
TPR	Protein-Protein interactions, Assembly of multi-protein complexes	AtCYP40/CYP40	BnCYP40-1	GmCYP8	GaCYP40-1	MtCYP40A	OsCYP40-1a	TaCYP41-1-7D	hCYP-40/Cyp40	Lamb et al., 1995; Galat, 2004; Mainali et al., 2014; Kumari et al., 2015; Schiene-Fischer, 2015; Hanhart et al., 2017; Chen et al., 2019; Singh et al., 2019; Ge Q. et al., 2020
			BnCYP40-2	GmCYP9	GaCYP40-2	MtCYP40B	OsCYP40-1b	TaCYP41-2-7A		
				GmCYP16	GaCYP40-3		OsCYP40-2	TaCYP41-3-7B		
				GmCYP17	GaCYP41			TaCYP44-1-6A		
					GaCYP45			TaCYP44-3-6B		
					GrCYP40-1			TaCYP44-3-6D		
					GrCYP40-3					
					GrCYP42-1					
					GrCYP42-2					
					GrCYP43					
					GhCYP28-4					
					GhCYP30-2					
					GhCYP40-1					
					GhCYP40-2					
					GhCYP40-3					
					GhCYP41					
					GhCYP44-2					
					GhCYP45-1					
					GhCYP45-2					
					GhCYP46					
					GbCYP37-2					
					GbCYP39-4					
					GbCYP40-3					
					GbCYP41-2					
					GbCYP43-1					
					GbCYP43-2					
					GbCYP49-1					
TPR+ Zf-SCNM1+ SCNM1- acidic	Protein-Protein interaction, Protein-RNA interaction, RNA splicing	–	–	–	GbCYP66-2	–	–	–	–	Buchner et al., 2003; Howell et al., 2007; Mainali et al., 2014
WD40 repeat	Assembly of multi-protein complexes	AtCYP71	BnCYP70-1	GmCYP20	GaCYP70,	MtCYP71	OsCYP71a	TaCYP72-1-7D	hCYP-73/Cyp73	Neer et al., 1994; Galat, 2004; Davis et al., 2008; Mainali et al., 2014; Kumari et al., 2015; Schiene-Fischer, 2015; Hanhart et al., 2017; Chen et al., 2019; Singh et al., 2019; Ge Q. et al., 2020
			BnCYP70-2	GmCYP35	GbCYP58		OsCYP71b	TaCYP72-2-7A		
								TaCYP72-3-7B		
					GrCYP63					
					GhCYP70-1					
					GhCYP70-2					
U-box	Ubiquitination	AtCYP65	–	GmCYP18	GaCYP65	MtCYP65		TaCYP64-4-4A	–	Aravind and Koonin, 2000; Mainali et al., 2014; Kumari et al., 2015; Chen et al., 2019; Singh et al., 2019; Ge Q. et al., 2020
				GmCYP19	GrCYP65			TaCYP64-5-4B		
					GhCYP65-1			TaCYP64-6-4D		
					GhCYP65-2					
U-box+Zf	Ubiquitination	–	BnCYP65-1	–	–	–	–	–	hCYP-58/Cyp60/Cyc4	Freemont et al., 1991; Lovering et al., 1993; Galat, 2004; Schiene-Fischer, 2015; Hanhart et al., 2017
			BnCYP65-2						hCYP-58i/Cyp60/Cyc4	
PsbQ-like	Plant specific oxygen evolving enhancer protein 3	–	BnCYP47-2	–	–	–	–	–	–	Balsera et al., 2003; Hanhart et al., 2017
			BnCYP47-3							
RRM	Regulation of transcription	–	–	–	–	MtCYPE-like	OsCYP59-1	TaCYP53-1-4B	hCYP-33/Cyp33/CYPE	Krzywicka et al., 2001; Galat, 2004; Kumari et al., 2015; Schiene-Fischer, 2015; Singh et al., 2019; Ge Q. et al., 2020
							OsCYP59-2	TaCYP54-1-4A		
								TaCYP55-1-4D	hCYP-57	
RRM + Zf	RNA splicing	AtCYP59	–	GmCYP56	GrCYP72-1	–	–	TaCYP37-1-3D	–	Mainali et al., 2014; Kumari et al., 2015; Yoshida et al., 2015; Chen et al., 2019; Singh et al., 2019
				GmCYP59	GhCYP70-3			TaCYP38-1-3B		
					GhCYP70-4			TaCYP45-1-3A		
					GbCYP47-1			TaCYP64-1-7A		
					GbCYP79			TaCYP64-2-7B		
								TaCYP64-3-7D		
Helical bundle	Signal transduction	AtCYP38/CYP38	–	–	–	–	–	–	–	Ulrich and Zhulin, 2005; Vasudevan et al., 2012
TPR+ RanBD1 + ZfRanBP + E3 SUMO Ligase	RanBD1/ZfRanBP: GTPase Ran binding	–	–	–	–	–	–	–	hCYP-358/Cyp358/RanBP2	Schiene-Fischer, 2015
	E3 SUMO Liagse: SUMO1 specific E3 ligase activity									
RRM+Zf+ R/K/E-rich + ATPase		–	BnCYP112	–	–	–	–	–	–	Yoshida et al., 2015; Hanhart et al., 2017
RRM+Zf+ Rho motif	–	–	–	–	–	MtCYP59A	–	–	–	Ge Q. et al., 2020
						MtCYP59B				
Transmembrane + Fip1 motif		–	BnCYP146	–	–	–	–	–	–	Askwith and Kaplan, 1997; Helmling et al., 2001; Hanhart et al., 2017
Coiled coil + S/K-R/E rich	–	–	BnCYP52	–	–	–	–	–		Liu et al., 2006; Weighardt et al., 1999; Hanhart et al., 2017
			BnCYP55							
Coiled coil	–	–	–	–	GaCYP47	–	–	–	–	Liu et al., 2006; Chen et al., 2019
					GrCYP47					
					GhCYP47					
					GhCYP48					
					GbCYP40-2					
					GbCYP61					
F-box	–	–	–	–	–	–	–	TaCYP23-2-6B	–	Craig and Tyers, 1999; Singh et al., 2019
								TaCYP26-1-6B		
								TaCYP26-6-6A		
PAN_4 domain	–	–	–	–	–	Medtr7g 081200	–	TaCYP34-1-5A	–	McMullen et al., 1991; Singh et al., 2019; Ge Q. et al., 2020
						Medtr5g 013540		TaCYP34-2-U		
								TaCYP35-1-4B		
Transposase_ Associated + Transposase Family tnp2	–	–	–	–	–	–	OsCYP 124	–	–	Majorek et al., 2014; Kumari et al., 2015
AAA +AAAlid3	Adenosine Tri Phosphatase (ATPase)	AtCYP67-1a	–	–	–	–	–	–	–	Confalonieri and Duguet, 1995; Neuwald et al., 1999; Kumari et al., 2015; Miller and Enemark, 2016
		AtCYP67-1b								
		AtCYP67-1c								
POP1 + POPLD + TR	–	–	–	–	GbCYP142	–	–	–	–	Lygerou et al., 1996; Chen et al., 2019
Herpes_ ICP4_C	–	–	–	–	–	MtCYP95A	–	–	–	Bruce and Wilcox, 2002; Ge Q. et al., 2020
						MtCYP95B				
Borrelia_P83	–	–	–	–	–	MtCYP57	–	–	–	Ge Q. et al., 2020

*The nomenclature of genes and proteins used is according to the respective publications. AAA, ATPase family associated with various cellular activities; AAA_lid_3, Alpha helical AAA+ lid domain located to the C-terminus of AAA domains; ATPase, an actin-like ATPase domain; Borrelia_P83, borrelia P83/P100 antigen proteins; CPSF1, Cleavage and polyadenylation specification factor subunit-1; E3 SUMO ligase domain; F-box, F-box domain; Fip1, Factor interacting with PAPOLA and CPSF1; FKBP, FK506 binding protein; Helix bundle domain, Four-Helix Bundle; Herpes_ICP4_C, The immediate-early protein ICP4 (Infected-cell polypeptide 4); PAN, PAN module; PAPOLA, Poly (A) polymerase alpha; POP1, Processing of precursor 1; POPLD, Processing of precursor 1(POP1)-like nuclear proteins; PPIL2, Peptidyl prolyl isomerase cis-trans isomerase-like 2; PPIL4, Peptidyl-prolyl cis-trans isomerase-like 4; PPWD1, Peptidyl prolyl isomerase domain and WD repeat-containing protein 1; PsbQ, Photosystem b Q (an extrinsic subunit of Photosystem II); RAN, Ras-related nuclear protein; RanBD1, Ran binding protein 1 domain; zf-RanBP, Zn-finger in Ran-binding proteins and others; RRM, RNA recognition motif; R/K/E-rich, a positively charged region (arginine, lysine, glutamate); S/K-R/E rich, Ser/Lys-Arg/Glu-rich region; SCNM1, Sodium channel modifier 1; SCNM1-acidic, Acidic c-terminal region of sodium channel modifier 1 SCNM1; SUMO, small ubiquitin-like modifier; TPR, Tetratricopeptide repeat; U-box, U-box domain; WD, Tryptophan-Aspartate repeat; TNP2, Nuclear transition protein 2; Zf, Zinc finger; Zf-SCNM1, Zinc finger of sodium channel modifier 1.*

So far, only five different plant cyclophilins *viz*., TaCYPA-1 (Sekhon et al., 2013), CsCYP (Campos et al., 2013), *Catharanthus roseus* Cat r 1 (Ghosh et al., 2014), BnCYP19-1 (Hanhart et al., 2019) and AtCYP38 or CYP38 (Vasudevan et al., 2012) have been characterized for their crystal structures. While the former four are single-domain proteins and show PPIase activity, the AtCYP38 is a MD cyclophilin that lacks *cis-trans* isomerization capability (Vasudevan et al., 2012). The crystal structures of TaCYPA-1, CsCYP, BnCYP19-1 and CLD of AtCYP38 are similar to “archetypal” human cyclophilin hCYPA, and consist of eight-stranded antiparallel β-barrel capped at either end by two α-helices (Vasudevan et al., 2012; Campos et al., 2013; Sekhon et al., 2013; Hanhart et al., 2019). However, Cat r 1 (PDB: 2MC9) shows variability in its structure since the β-barrel in this protein consists of seven antiparallel β-strands instead of eight (Ghosh et al., 2014). The CsA-binding site in hCYPA and other such cyclophilins is composed of seven aromatic and other hydrophobic residues that constitute the hydrophobic core within the barrel (Kallen et al., 1991). The topology of this β-barrel structure is unique in the sense that it remains occupied with a set of closely packed aromatic groups making no room for binding of either CsA or the Pro containing peptides (Ke, 1992). Therefore, the CsA and other substrates bind to an active site that is formed by amino acid residues located on the outer face of the β sheet. The active sites consist of 13 residues which are identical in CsCYP, TaCYPA-1, BnCYP19-1 and hCYPA (Ke et al., 1994; Campos et al., 2013; Sekhon et al., 2013; Hanhart et al., 2019). However, the electrostatic surface map studies indicated that despite conservation of all the 13 active site residues, the active site pocket in Cat r 1 appears to be slightly broader and is more acidic in nature, which might be imparting precision for binding of peptides with a specific amino acid composition (Ghosh et al., 2014). While the conservation of CLD structure in cyclophilins underlines its fundamental role in the cell, the remarkable diversity in their domain architecture could have subtle or profound effects on the structure of these proteins which may, in turn, affect their biochemical activities differently, enabling them to perform a wide variety of roles in different cellular processes. Elucidation of crystal structures of different cyclophilins and identification of their interacting proteins is, thus, imperative to gain further insights into their specific functions.

## Regulation of PPIase Activity of Cyclophilins

The PPIase activity of immunophilins is assayed by several *in vitro* methods *viz*., isomer-specific cleavage of the peptide with chymotrypsin, protease-free assay, NMR-based methods, protein folding/unfolding and fluorescence-based assays (Fischer et al., 1984; Janowski et al., 1997; Davis et al., 2010). The recent development of an *in vivo* method provides a useful tool to study the regulation of PPIase activity by temporal, spatial and environmental factors in the living cells (Jiang et al., 2018). Cyclophilins have been characterized biochemically from several organisms (Table 6), some of which were reviewed earlier (Fanghänel and Fischer, 2004). As observed for cyclophilins in other organisms, the plant cyclophilins also exhibit variability in their kinetic parameters and sensitivity to CsA (Table 6). Whereas the catalytic constants (k_cat_/k_m_) of the different plant cyclophilins reported until now vary between 10^5^ to 10^7^ M ^–1^s^–1^ for the suc-AAPF-pNA oligopeptide substrate, the inhibition constants for CsA range between 6.0 (ZmCYP18) to 78.3 nM (TaCYPA-1). The implications of diversity in biochemical attributes of cyclophilins in modulating the physiological response in plants are not understood and need to be investigated by overexpressing mutant cyclophilins that exhibit graded *cis-trans* isomerization capabilities.

**TABLE 6:** Biochemical characteristics of different cyclophilins.

Source	Cyclophilin	PPIase Activity		Chaperonic activity	References
		
		Catalytic efficiency (k_cat_/k_m;_M^–1^s^–1^)	Inhibition constant (K_i_) for CsA (nM)		
**Plants**					
*Arabidopsis thaliana*	AtCYP19-3/ROC2^a,1^	4.88x10^6^	18.75	NA	Romano et al., 2004b; Kaur et al., 2015
	AtCYP19-4/CYP5^a,1^	5.7x10^6^	8.0	NA	Romano et al., 2004b; Grebe et al., 2000
	AtCYP20-3/ROC4^b,1^	8.32x10^6^	CsA inhibitable	NA	Motohashi et al., 2003; Romano et al., 2004b
	AtCYP38/CYP38	PPIase inactive	–	–	Vasudevan et al., 2012
*Brassica napus*	BnCYP18-4^a,1^	9.02 x10^6^	14.2	NA	Hanhart et al., 2019
	BnCYP18-5^a,1^	5.30x10^6^	22.4	NA	Hanhart et al., 2019
	BnCYP19-1^a,1^	9.07x10^6^	16.6	NA	Hanhart et al., 2019
*Citrus sinensis*	CsCYP^a,1^	5.6x10^6^	NA	NA	Campos et al., 2013
*Oryza sativa*	OsCYP2^a,1^	4.5x10^6^	NA	NA	Kumari et al., 2009
*Ricinus communis*	RcCYP1^a,1^	9.48x10^6^	NA	NA	Gottschalk et al., 2008
*Spinach oleracea*	TLP40^a,1^	1.6x10^6^	CsA insensitive	NA	Fulgosi et al., 1998
	TLP20 ^a,1^	NA	CsA inhibitable	NA	Edvardsson et al., 2003
*Triticum aestivum*	TaCYPA-1^a,1^	2.32x10^5^	78.3	NA	Sekhon et al., 2013
*Vicia faba*	pCYPB^a,1^	NA	3.9	NA	Luan et al., 1994
*Zea mays*	Cytosolic PPI^a,1^	1.1x10^7^	6.0	NA	Sheldon and Venis, 1996
	Microsomal PPI^a,1^	25x10^6^	6.0	NA	Sheldon and Venis, 1996
**Animals**					
Bovine	CYP^a,1^(Bovine cyclophilin)	1.3 x10^7^	45 ± 3	NA	Kofron et al., 1991
	ERPPI^a,1^	3.0 x10^6^	5.0	NA	Bose et al., 1994
*Drosophila melanogaster*	Moca-CYP^a,1^	5.6x10^4^	450.0	NA	Cavarec et al., 2002
*Homo sapiens*	CYPA^a,1^	1.4x10^7^	19	NA	Liu et al., 1990
	hCYPB/hCYP-22^a,1^	6.3x10^6^	6.9	NA	Roydon Price et al., 1991
	hCYPD/CYP-40^a,1^	1.9x10^6^	300.0	Observed	Kieffer et al., 1992; Freeman et al., 1996
	CYP18^a,1^	5.6x10^–6^	1.5	Observed	Janowski et al., 1997; Moparthi et al., 2010
	NK-CYP^a,1^	7.4x10^5^	770.0	Observed	Rinfret et al., 1994
*Rattus norvegicus*	Matrin CYP^a,1^	1.0x10^6^	220.0	NA	Mortillaro and Berezney, 1998
	PPIase^a,1^	0.9x10^6^	3.6	NA	Connern and Halestrap, 1992
*Tachypleus tridentatus*	CYPG^b,1^	1.8x10^5^	8.3	NA	Takaki et al., 1997
*Xenopus laevis*	XlCYP	1.1x10^7^	NA	NA	Miele et al., 2003
**Protozoa**					
*Plasmodium falciparum*	PfCYP19A^a,1^ PfCYP19B^a,1^	6.3x10^6^ 5.7x10^6^	10 15	Observed Observed	[Bibr B168][Bibr B168]
	PfCYP^a,1^	2.3x10^6^	10.0	NA	Hirtzlin et al., 1995
**Fungi**					
*Aspergillus nidulans*	CYPB^a,1^	PPIase active	3.0	NA	Joseph et al., 1999
*A. niger*	CYPA	PPIase active	NA	NA	Derkx and Madrid, 2001
*Candida albicans*	CYP1	PPIase active	NA	NA	Koser et al., 1990
*Neurospora crassa*	NcCYP41^a,1^	6.5x10^5^	7.0-8.0	NA	Faou, 2001
	NcCYP-19^a,1^	2.8x10^6^	NA	NA	Schonbrunner et al., 1991; Galat, 1999
*Saccharomyces cerevisiae*	yCYPA/CPR1^a,1^	1.52x10^7^	40.0 ± 8	NA	Zydowsky et al., 1992a
	yCYPB/CPR2^a,1^	5.77x10^6^	101.0 ± 14	NA	Zydowsky et al., 1992a
	CPR3^a,1^	5.8x10^6^	CsA inhibitable	NA	Scholze et al., 1999
	CPR6^a,1^	4.8x10^5^	CsA inhibitable	Observed	Mayr et al., 2000
	CPR7^a,1^	7.5x10^4^	CsA inhibitable	Observed	Mayr et al., 2000; Kumar et al., 2015
*Schizosaccharomyces pombe*	SpCYP3^a,1^	1.5x10^6^	NA	NA	Pemberton et al., 2003
**Bacteria**					
*Bacillus subtilis*	PPiB^a,1^	1.1 x10^6^	120.0	NA	Achenbach et al., 1997
*Escherichia coli*	PPIA^b,1^	5.71x10^7^	25000-50000	NA	Compton et al., 1992
	PPIB^b,1^	6.74x10^7^	25000-50000	NA	
*Legionella pneumophila*	LpCYP18	4.6x10^6^	NA	NA	Schmidt et al., 1996
*Streptomyces antibioticus*	SanCYP18^a,1^	7.92 x10^6^	21000	NA	Manteca et al., 2004
*Streptomyces chrysomallus*	ScCYPA^a,1^	3.73x10^6^	25.0	NA	Pahl et al., 1992
	ScCYPB^a,1^	7.5x10^6^	75.0	NA	Pahl et al., 1997
**Nematode**					
*Caenorhabditis elegans*	CYP1^a,1^	7.0x10^4^	NA	NA	Page et al., 1996
	CYP2^a,1^	6.1x10^5^	NA	NA	Page et al., 1996
	CYP3^a,1^	3.6x10^5^	NA	NA	Page et al., 1996
	CYP4^a,1^	1.8x10^4^	NA	NA	Page et al., 1996
	CYP5^a,1^	7.4x10^4^	NA	NA	Page et al., 1996
	CYP6^a,1^	8.4x10^6^	NA	NA	Page et al., 1996
	CYP8^a,1^	1.95x10^4^	NA	NA	Page et al., 1996
	CYP9^a,1^	1.5x10^4^	NA	NA	Page et al., 1996
	CYP10^a,1^	1.9x10^4^	NA	NA	Page et al., 1996
	CYP11^a,1^	1.5x10^4^	NA	NA	Page et al., 1996
**Other organisms**					
*Brugia malayi*	BmCYP1^a,1^	7.9 x10^6^	860.0	NA	Page et al., 1995
	BmCYP2^a,1^	1.23x10^7^	9.3	NA	Ma et al., 1996
*Dictyostelium discoideum*	CYPE^a,1^	PPIase active	NA	NA	Skružný et al., 2001
*Leishmania major*	LmCYP19^a,1^	1.5x10^6^	5.2	NA	Rascher et al., 1998
*Schistosoma mansoni*	SmCYPB^a,1^	8.2x10^5^	20.0	NA	Bugli et al., 1998
	SmCYPA^a,1^	3.65x10^5^	72.0	NA	Bugli et al., 1998
*Toxoplasma gondii*	CYP18.5^a,1^	NA	32.0	NA	High et al., 1994
	CYP20^a,1^	NA	5.0	NA	High et al., 1994
*Trypanosoma cruzi*	TcCYP19^a,1^	NA	18.4	NA	Búa et al., 2001

*The nomenclature of proteins used is according to the respective publications. a, N-succinyl-Ala-Ala-Pro-Phe-p-nitroanilide as substrate; b, N-succinylAla-Ala-Pro-Phe-4-methylcoumaryl-7-amide as substrate; 1, Chymotrypsin used as proteolytic enzyme; CsA, Cyclosporin A; NA, Information not available.*

Contingent upon the presence of an extra loop of four or more amino acid residues present at residue 50 corresponding to hCYPA, the cyclophilins are classified as divergent or non-divergent (Dornan et al., 1999). The divergent loop cyclophilins such as TaCYPA-1 (Sekhon et al., 2013), CsCYP (Campos et al., 2013) and Cat r 1 (Ghosh et al., 2014) are similar to hCYPA in their active site composition and CsA binding characteristics except for the presence of a characteristic additional loop (consensus sequence XXGKXLH corresponding to amino acid residues 48–54 in TaCYPA-1), two conserved Cys residues (Cys40 and Cys168) and a conserved glutamate (Glu83) residue (Kaur et al., 2015; Vasudevan et al., 2015). On the contrary, the non-divergent cyclophilins such as hCYPA, SmCYPA and AtCYP20-3 or ROC4 (Rotamase Cyclophilin 4) lack the additional loop and are characterized by two conserved Cys residues at positions 122 and 126 (Gourlay et al., 2007; Laxa et al., 2007). AtCYP38, however, is a unique kind of non-divergent cyclophilin since it lacks both the characteristic divergent loop as well as the Cys amino acids observed in other plant non-divergent cyclophilins (Vasudevan et al., 2012).

The PPIase activity of cyclophilins, in general, is regulated in a redox-dependent or independent manner. Contrary to the *E. coli* cyclophilin PPIB, that is regulated by redox-independent mechanisms (Hayano et al., 1991; Kaur et al., 2015), the PPIase activity of AtCYP19-3 (ROC2), AtCYP20-3, SmCYPA, CsCYP and TaCYPA-1 is subject to redox regulation (Motohashi et al., 2003; Gourlay et al., 2007; Laxa et al., 2007; Campos et al., 2013; Kaur et al., 2015, 2017). Furthermore, the redox-regulatory mechanisms observed in different cyclophilins are also distinct. For instance, the regulation of non-divergent cyclophilins hCYPA and AtCYP20-3 involves glutathionylation and thioredoxin-mediated thiol-disulfide exchange, respectively. Whereas glutathionylation of Cys residues in hCYPA renders the protein inactive under oxidative conditions, deglutathionylation through reduction of thiol groups by intracellular pH changes or in response to reducing environment restores its activity (Ghezzi et al., 2006; Townsend, 2007; Dalle-Donne et al., 2009). On the contrary, the activity of AtCYP20-3 is modulated by thioredoxin (Trx)-mediated thiol-disulphide exchange (Motohashi et al., 2003; Laxa et al., 2007). Under oxidizing conditions, the formation of two disulphide pairs in AtCYP20-3 (Cys53-Cys70 and Cys128-Cys175) abrogates the PPIase activity, while Trx-mediated reduction results in restoration of the catalytic function.

Regulation of another non-divergent cyclophilin SmCYPA from *Schistosoma mansoni* is attributed to oxidation-induced disulfide bond formation between Cys122 and Cys126 that results in loss of activity (Gourlay et al., 2007). On the contrary, the regulation of a divergent cyclophilin from *Citrus sinensis*, CsCYP, involves both disulphide bond formation between Cys40 and Cys168 as well as loop displacement (Campos et al., 2013). Our earlier studies revealed that the wheat divergent cyclophilin, TaCYPA-1, has an additional Cys126 residue corresponding to the residue 126 in non-divergent SmCYP (Gourlay et al., 2007; Kaur et al., 2015). Site-directed mutagenesis studies provided evidence that PPIase activity of TaCYPA-1 is regulated through a dual mechanism involving loop displacement (Kaur et al., 2017), as observed in the divergent cyclophilin CsCYP (Campos et al., 2013), and also by the interaction between Cys122 and Cys126, as reported for the non-divergent SmCYPA (Supplementary Figure 1; Gourlay et al., 2007), with the latter mechanism playing a predominant role (Kaur et al., 2017). These observations make TaCYPA-1 unique since despite being a divergent cyclophilin its activity is also subject to regulation by mechanisms that are more common to the non-divergent cyclophilins. *In silico* studies in our lab revealed that several other wheat cyclophilins may also follow similar regulation (Singh et al., 2019), the significance of which is not understood yet. It is evident that despite the conservation of active sites in cyclophilins, distinct regulatory mechanisms have evolved for the regulation of these proteins, possibly to impart versatility to these proteins to regulate diverse cellular processes. However, the physiological implication of different regulatory mechanisms of cyclophilins in plants is a matter of conjecture and merits further investigations.

## Cyclophilins as Protein Folding Catalysts

Evidence for *in vivo* role of cyclophilins in protein folding was first provided by analysis of *Drosophila melanogaster* ninaA (Neither inactivation nor after potential protein A) protein, which is a tissue-specific integral membrane protein required for the proper synthesis of the visual pigment rhodopsin 1 (Rh1; Stamnes et al., 1991). In *D. melanogaster*, Rh1 is synthesized in the ER and is transported to rhabdomeres via the secretory pathway where it performs phototransduction. Mutation in ninaA blocks this transportation and results in accumulation of rhodopsin in the ER, leading to its degradation and consequently impaired visual function (Colley et al., 1991). The CPR3 in yeast also catalyzes protein folding *in vivo*, as isolated mitochondria from Δ*cpr3* (yeast strain mutated in *CPR3* gene) showed a reduced rate of protein folding (Matouschek et al., 1995). The chaperonic function of an *Arabidopsis* cyclophilin AtCYP40 (CYP40) was shown to be independent of PPIase activity since the enzymatically inactive mutants of AtCYP40 were able to facilitate the assembly of RNA induced silencing complex (RISC; Iki et al., 2012). Evidence for the chaperonic role of RcCYP1, a highly active PPIase abundant in companion cell sieve element complex of *Ricinus communis*, was provided by microinjection studies (Gottschalk et al., 2008). These authors observed that RcCYP1 is involved in auto-cell to cell trafficking via interaction with plasmodesmata special proteins and performs unique functions by assisting their refolding. Studies carried out in our laboratory demonstrated that PPIase activity in the wheat grains is associated with the deposition of grain storage proteins or prolamines (Dutta et al., 2011). Since prolamines are rich in prolyl residues (10–15%; Shewry et al., 2002), the PPIases might be involved in the folding of these proteins. Plants have diverse cyclophilins, but information on biochemical properties and chaperonic activities of these proteins is rather scarce. Therefore, molecular analysis and biochemical characterization of different cyclophilins in plants are imperative for gaining insights into their physiological roles which might further lead to the development of crops with improved agronomic traits.

## Roles of Cyclophilins in Chloroplast

The CsA-sensitive PPIase activity in chloroplasts was first demonstrated in pea by Breiman et al. (1992). Since the characterization of TLP40, a 40 kDa thylakoid lumen cyclophilin from spinach chloroplasts (Fulgosi et al., 1998), proteomics and bioinformatics approaches resulted in the identification of 11 FKBPs and 5 cyclophilins in the chloroplast lumen of *Arabidopsis* (Edvardsson et al., 2007; Trivedi et al., 2012). TLP40 is a multi-domain cyclophilin that shows PPIase activity and acts as a negative regulator of the thylakoid membrane protein phosphatase (Fulgosi et al., 1998; Vener et al., 1999). This protein plays an essential role in the growth and development of plants since mutations in its *Arabidopsis* ortholog, AtCYP38, resulted in impaired development of chloroplasts, retarded plant growth, hypersensitivity to light, and enhanced degradation of D1 and D2 components of PSII under high light conditions (Fu et al., 2007; Sirpiö et al., 2008; Vasudevan et al., 2012; Vojta et al., 2019). Together with other immunophilins such as FKBP13 and FKBP20-2, that are required for accumulation of the cytochrome b6f complex and PSII supercomplexes, respectively (Gupta et al., 2002; Lima et al., 2006), AtCYP38, despite lacking PPIase activity, appears to be indispensable for proper biogenesis and maintenance of photosynthetic complexes. On the contrary, impaired functioning of AtCYP20-2, a highly active PPIase and orthologous to the spinach cyclophilin TLP20, had no apparent phenotypic effect, suggesting redundancy in the function of these proteins (Fulgosi et al., 1998; Sirpiö et al., 2009). It has been proposed that while TLP40 performs specialized regulatory function(s), TLP20 might act as a general protein folding catalyst (Edvardsson et al., 2003). The chloroplast stromal protein AtCYP20-3, 65.64 % identical to AtCYP20-2, facilitates the folding of serine acetyltransferase (SAT) that catalyzes the ultimate step in Cys biosynthesis which is important for glutathione formation. The PPIase and folding activities of AtCYP20-3, sensitive to photooxidation and stress-induced ROS, were restored following reduction by photoreduced Trx (Laxa et al., 2007). Mutation in *AtCYP20-3* resulted in hypersensitivity to oxidative stress in *Arabidopsis* (Dominguez-Solis et al., 2008), implying that it enables the Cys-based thiol biosynthesis pathway to adjust to light and stress conditions. Isothermal titration microcalorimetry and gel overlay assays further indicated that AtCYP20-3 interacts with thiol based peroxidases, 2-Cysteine peroxiredoxins (2-CysPrx), which can exist as either dimer or decamer. The dimer form is favored under oxidizing conditions whereas the decamer is formed under reducing conditions. High affinity of AtCYP20-3 for the dimer leads to a decrease in the free dimer concentration. Thus it appears that AtCYP20-3 regulates the critical transition concentration (concentration responsible for dimer-decameric form transition) value of 2-CysPrx, suggesting redox-dependent conformational dynamics of this protein (Liebthal et al., 2016).

## Roles of Cyclophilins in Growth and Development of Plants

Various studies have substantiated the role of cyclophilins in the regulation of different aspects of plant growth and development. Whereas, a CsA-inhibitable PPIase in *Arabidopsis*, AtCYP19-4 (CYP5), was proposed to determine cell-polarity and regulate embryogenesis, the cytosolic SD cyclophilin AtCYP19-1 (ROC3) was implicated in seed development (Grebe et al., 2000; Stangeland et al., 2005). Cyclophilins also appear to affect organogenesis in *Arabidopsis* since the loss of function of a nuclear-localized MD protein, AtCYP71, resulted in compromised lateral organ formation and apical meristem activity (Li et al., 2007). Chromatin remodeling and transcriptional regulation were proposed as the likely mechanisms of action for AtCYP71 because this protein exhibited interaction with FAS1 (a subunit of Chromatin Assembly factor-1) and LHP1 (a heterochromatin protein) (Li et al., 2007; Li and Luan, 2011).

Another cytosolic cyclophilin, AtCYP40, was identified as a regulator of vegetative growth in *Arabidopsis*. Mutation (*sqn*) in this gene (*SQUINT*) resulted in a decrease in the number of juvenile leaves (Berardini et al., 2001). The mutated plants exhibited attenuated ARGONAUTE1 (AGO1) function that decreased the miRNA activity, resulting in enhanced expression of miR156-sensitive squamosa promoter binding protein-like family (SPL) of transcription factors (Smith et al., 2009). Even though reproductive maturation was not affected in the *sqn* mutants, later studies revealed that AtCYP40, along with REBELOTE (RBL; protein of unknown function) and ULTRAPET ALA (ULT1; a putative transcription factor), is important for floral developmental homeostasis (Prunet et al., 2008). AtCYP40 is a multidomain cyclophilin and contains TPR domain at its C-terminus which mediates its interaction with cytoplasmic HSP90, a feature also conserved for its orthologs in animals and *S. cerevisiae* (Berardini et al., 2001; Wandinger et al., 2008; Earley and Poethig, 2011; Blackburn et al., 2015). AtCYP40 facilitates the formation of miRISC assembly by mediating the interaction of HSP90-AGO1 complex with a small RNA duplex that leads to the formation of mature RISC. Though the interaction of AtCYP40 with HSP90-AGO 1 complex, imperative for RISC assembly, is sensitive to CsA, the role of PPIase activity in this process is still elusive (Iki et al., 2012).

Recent studies have demonstrated that regulation of growth and development in plants by cyclophilins may also be isoform-dependent (Jung et al., 2020). The Golgi-localized cyclophilin in rice, OsCYP21, exists in four different isoforms *viz*., OsCYP21-1, OsCYP21-2, OsCYP21-3 and OsCYP21-4. Despite the conservation of active site residues, these isoforms differ in their activity. While OsCYP21-1 and OsCYP21-2 are enzymatically active, the latter two lack PPIase activity. The isoforms OsCYP21-1 and OsCYP21-2 were implicated in the regulation of growth and development through modulation of ABA pathway genes. The significance of PPIase activity in this role needs to be corroborated by generating plants with mutated OsCYP21-1 and OsCYP21-2 that are deficient in PPIase function. Thus, it is evident that the regulation of various facets of growth and development by different cyclophilins entails distinct mechanisms that further signifies their functional versatility.

### Implications of Cyclophilins in Hormone Signaling

Recent studies have provided evidence for the involvement of cyclophilins in several hormone-mediated responses in plants. Brassinosteroids and gibberellic acid (GA) are key regulators of plant stem elongation, and defects in the biosynthetic or signaling pathways of these hormones result in dwarf phenotype (Wang and Li, 2008). Genes contributing to dwarfness are of agronomic importance due to their potential for developing crops that are resistant to lodging under water-logging and strong wind conditions. DELLA proteins (named after conserved N-terminal D-E-L-L-A amino acid sequence) are inhibitors of stem growth and have been implicated in dwarf phenotype in *Arabidopsis*, *B. napus* and peach (Lawit et al., 2010; Zhao et al., 2017; Cheng et al., 2019). GA degrades DELLA proteins via the ubiquitin-proteasome pathway to promote stem growth (Sun, 2008, 2010). Mutations in the DELLA domain that abrogate interaction with F-box containing proteins SLY1, GID1 and GID2 prevent their GA-dependent degradation (Dill et al., 2004; Ueguchi-Tanaka et al., 2005; Nakajima et al., 2006; Lou et al., 2016). Functional impairment of DELLA proteins was reported to result in the dominant GA-insensitive dwarf phenotype (*gaid*) in wheat and *B. rapa* (*Brrga1-d*) (Ho et al., 1981; Muangprom et al., 2005). The *gaid* phenotype in wheat was also associated with higher levels of a 20 kDa cyclophilin, TaCYP20-2, overexpression of which in the wild-type wheat lead to *gaid*-like phenotype (Li et al., 2010), implying that this protein plays an essential role in maintaining GA homoeostasis by regulating the DELLA proteins. However, elucidation of the precise mechanism of action requires further intense experimentations.

The inhibition of hypocotyl growth and the expansion of cotyledons by light after the emergence of shoot from the soil in *Arabidopsis* is regulated by the photoreceptors phytochromes (PHYA to PHYE) and cryptochromes (CRY1 and CRY2) (Cashmore et al., 1999; Quail, 2005). Screening of the transgenic *Arabidopsis* 35S-cDNA lines for defective de-etiolation under a combination of blue and far-red light resulted in the isolation of a mutant (*roc1-1D*) that depicted enhanced expression of a cytoplasmic cyclophilin, AtCYP18-3 (ROC1, Rotamase Cyclophilin 1). The *roc1-1D* plants exhibited long hypocotyls and poorly unfolded cotyledons under blue and far-red light, and lower anthocyanin under far-red or blue light (Trupkin et al., 2012). Further analysis revealed that the mutant plants were hypersensitive to brassinosteroids in light but not in the dark. Inhibition of brassinosteroid synthesis and mutations in the genes responsible for brassinosteroid signaling abolished the mutant phenotype, implying that AtCYP18-3 links cryptochrome and phytochrome to brassinosteroid sensitivity (Trupkin et al., 2012).

Subsequent studies also provided evidence that functionality of AtCYP18-3 is highly sensitive to single amino acid substitution, since plants which over-expressed its variant containing phenylalanine instead of serine at position 58 exhibited reduced height, increase in shoot branching and higher sensitivity to photoperiod and temperature (Ma et al., 2013). The wild type AtCYP18-3 though does not appear to control stem elongation, likely conformation changes due to amino acid substitution might have resulted in the identification of new targets, thereby, affecting the stem growth. Therefore, structural analysis and identification of interacting proteins are imperative to understand the molecular mechanisms by which the mutated AtCYP18-3 controls growth and development in plants. Further, whether the mutated AtCYP18-3 can facilitate cross-talk between brassinosteroid signaling and photoreceptors is also a subject of future studies.

**FIGURE 2:**
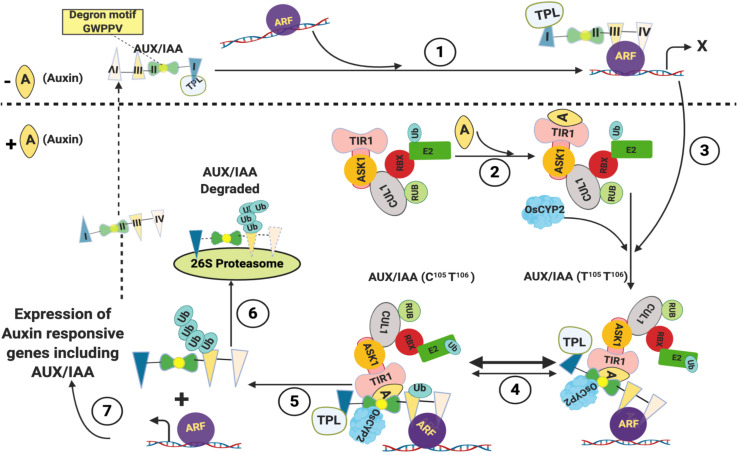
Role of cyclophilins in regulation of Auxin-responsive genes. At low levels of Auxin, Aux/IAA proteins bind to auxin response factors (ARFs) directly or through recruitment of transcriptional corepressor such as TOPLESS (TPL) and inhibit their activity (1). When present at high levels, the auxin binds to its receptor TRANSPORT INHIBITOR RESPONSE1 (TIR1) and enhances its interaction with the highly conserved ‘degron’ motif GWPPV in domain II of Aux/IAAs (2 and 3). The Aux/IAA proteins bind to SCFTIR1-Auxin complex only when W104-P105 isomer in the ‘degron’ motif GWPPV (residues 103, 104, 105, 106 and 107, respectively, in rice) is in cis conformation. The trans conformer of W-P (T105-T106) in the ‘degron’ motif is catalyzed to cis form (c105-T106) by OsCYP2 (4). The Aux/IAA-SCFTIR1 complex leads to ubiquitination of Aux/IAA proteins (5), which are then degraded by 26S Proteasome (6), leading to expression of Auxin responsive genes including Aux/IAA (7) (Adapted from Tan et al., 2007; Mockaitis and Estelle, 2008; Jing et al., 2015; Created with BioRender.com).

Besides brassinosteroid and GA signaling, cyclophilins have also been demonstrated to mediate auxin response. At low levels of auxin, the expression of auxin-responsive genes is kept in check by the unstable transcriptional repressors Aux/IAA proteins that bind to and inhibit the activity of auxin response factors (ARFs), a family of transcriptional activators (Figure 2; Theologis et al., 1985; Ainley et al., 1988; Conner et al., 1990; Yamamoto et al., 1992; Guilfoyle et al., 1993; Abel et al., 1995). The Aux/IAA genes are also induced by IAA and control the auxin response through a negative feedback loop (Reed, 2001). The Aux/IAA proteins consist of four highly conserved domains I-IV and bind to the ARFs either directly or through recruitment of transcriptional corepressor such as TOPLESS (TPL), the interactions being mediated by domain I that contains Leu-rich motif (Tiwari et al., 2004; Szemenyei et al., 2008). At high levels, the auxin binds to its receptor TRANSPORT INHIBITOR RESPONSE1/AUXIN SIGNALING F-BOX PROTEINS (TIR1/AFBs), an F-box containing protein, and the auxin-responsive genes are activated through auxin-dependent proteasomal degradation of Aux/IAA proteins that require ubiquitination (Wang and Estelle, 2014). The ubiquitination of proteins is catalyzed by a cascade of three enzymes *viz*., the Ub-activating enzyme (E1), the Ub-conjugating enzyme (E2) and the Ub-protein ligase (E3). The SCF (Skp1-Cul1-F box) E3, one of the four different types of E3s described in plants, is a complex of four different polypeptides viz., SKP1 (a member of an ASK family in plants), CDC53 or Cullin (Cul1), an F Box protein and RBX. The Cul1 acts as a central scaffold protein, while the SKP1 interacts with the F-box protein that further binds to the substrate proteins (Smalle and Vierstra, 2004). Transfer of Ub from Ub-E2 to the substrate protein is catalyzed by the fourth subunit (RBX1, ROC, or Hrt1) of the SCF complex (Petroski and Deshaies, 2005). The TIR1 interacts with SKP1 to form the SCF^*TIR1*^ complex (Ruegger et al., 1998; Gray et al., 1999). Auxin acts as a molecular glue and after binding to TIR1, it enhances the interaction of the latter with the highly conserved ‘degron’ motif GWPPV in domain II of Aux/IAAs, leading to ubiquitination and proteolytic degradation of the latter (Figure 2; Gray et al., 2001; Reed, 2001; Tan et al., 2007). The Aux/IAA proteins bind to SCF^*TIR1*^-Auxin complex only when the ‘degron’ motif GWPPV is in the *cis* W-P isomer (Tan et al., 2007; Acevedo et al., 2019). Recent studies have provided insights into the implications of cyclophilin-associated PPIase activity in mediating the interaction of Aux/IAA with the SCF^*TIR1*^-Auxin complex. The *LATERAL ROOTLESS 2* (*LRT2*) in rice encodes a cyclophilin PPIase OsCYP2, and disruption of this gene leads to an auxin-resistant phenotype and defective development of lateral roots (Kang et al., 2013; Zheng et al., 2013). The OsCYP2 was demonstrated to physically interact with the rice OsAux/IAA and TIR proteins, and catalyze the *cis-trans* isomerization of the OsIAA11 degron motif (Jing et al., 2015). These findings, thus, imply that the equilibrium of *cis* to *trans* populations of Aux/IAA proteins acts as a molecular timer to regulate auxin signal transduction (Acevedo et al., 2019). Since transcription of genes responsive to jasmonic acid, GA and strigolactone is also dependent on proteasome-mediated degradation of their specific repressors, the involvement of PPIases in controlling regulatory circuits of other hormones cannot be ruled out and should be the subject of future studies.

Given the diversity of PPIases in plants, it is likely that parallel regulatory mechanisms may be operating for several other processes in plants that, nonetheless, are yet to be identified. The presence of different functional domains, several of which facilitate protein-protein interactions, may enable the cyclophilins to identify a multitude of proteins as targets, thereby controlling complex regulatory circuits that enable the plants to respond to various developmental and environmental cues. It is apparent that, as proposed earlier for several biological processes such as cell division, gene expression, immune response and neural functions in animals (Lu et al., 2002, 2007), the PPIase catalyzed *cis-trans* conversion may act as a molecular switch in plants as well.

### Roles of Cyclophilins in Transcriptional and Post-transcriptional Gene Regulation

Transcript turnover and translational control are important post-transcriptional mechanisms of regulation of gene expression. Several cyclophilins have been reported to contain RNA Recognition Motif (RRM), a 90 amino acid long conserved RNA binding motif that is a characteristic feature of RNA-interacting proteins known to actively participate in pre-mRNA processing events (Kenan et al., 1991; Birney et al., 1993). This group of proteins is popularly known as cyclophilin-RNA interacting proteins (CRIPs). The first gene belonging to this group, *KIN241*, was identified in *Paramecium* and demonstrated to play an essential role in cell morphogenesis, cortical organization and nuclear reorganization (Krzywicka et al., 2001). The *Arabidopsis* cyclophilin AtCYP59, which besides PPIase domain also contains an RRM motif, a Zn-knuckle and a charged C-terminal domain consisting of RS/RD (arginine/serine and arginine/aspartate) repeats, was proposed to regulate transcription through its interaction with the immature mRNA (Gullerova et al., 2006; Bannikova et al., 2012). However, contrary to human RRM-containing cyclophilin hCYP33 (CYPE), that showed enhanced PPIase activity after binding to RNA (Wang et al., 2008), the catalytic activity of AtCYP59 was repressed by RNA, indicating a possible negative feedback loop. The physiological significance of this observation in plants is, however, still to be established. Though the presence of RRM along with other domains is also observed in other cyclophilins *viz.*, BnCYP52, BnCYP55 and BnCYP112 in *B. napus*, and TaCYP37-1-3D, TaCYP38-1-3B, TaCYP45-1-3A, TaCYP53-1-4B, TaCYP54-1-4A, TaCYP55-1-4D, TaCYP64-1-7A, TaCYP64-2-7B and TaCYP64-3-7D in wheat (Hanhart et al., 2017; Singh et al., 2019), the precise role of these proteins in RNA processing or transcriptional regulation is only speculative. A multi-domain cyclophilin, BnCYP146, the largest cyclophilin in *B. napus*, exhibits the presence of a putative Fip1 domain that has not been identified earlier in any of the cyclophilins. As Fip1 is a transmembrane motif involved in polyadenylation of mRNAs via interaction with the poly(A) polymerase (Hanhart et al., 2017), BnCYP146 might have a role in the stabilization of target RNA molecules and, hence, in the regulation of translation. This, however, requires further validation.

### Implications of Cyclophilins in Abiotic Stress Response

The expression of cyclophilins in plants and other organisms is regulated by several different stress conditions (Table 7), supporting their role in the adaptation process (Marivet et al., 1994; Godoy et al., 2000; Sharma et al., 2003; Sekhar et al., 2010; Kumari et al., 2013, 2015). Our studies on sorghum were the first in plants to demonstrate that stress-induced PPIase activity is associated with drought tolerance (Sharma and Singh, 2003a,b; Sharma et al., 2003). Since then, conclusive evidence for the role of cyclophilins in the adaptation of plants to abiotic stress has been provided by several transgenic studies (Table 7). Heterologous expression of pigeonpea (*CcCYP*) and Golgi-localized rice (*OsCYP21-4*) cyclophilins imparted tolerance against salt and oxidative stress in *Arabidopsis* and rice (*Oryza sativa*), respectively (Sekhar et al., 2010; Lee et al., 2015a). Ectopic expression of a cold-induced cyclophilin PPIase, *OsCYP19-4*, in transgenic rice resulted in a significant increase in the number of tillers, spikes, grain weight, and was associated with cold resistance (Yoon et al., 2016). Due to high similarity (70 %) to AtCYP19-4 (Ahn et al., 2010), that interacts with guanine nucleotide exchange factor (GNOM protein) which is involved in polar localization of the auxin efflux carrier PIN1, the enhanced performance of *OsCYP19-4* overexpressing plants was ascribed to alteration in auxin homeostasis (Yoon et al., 2016). Determination of the auxin levels is required to support the proposed mechanism.

**TABLE 7:** Abiotic stress modulated cyclophilin genes.

Organism	Cyclophilin gene	Accession no.	Activity	Role in Stress	References
**Plants**					
*Arabidopsis thaliana*	*AtCYP5*	NC_003071	PPIase activity	Cold and salt	Saito et al., 1999a; Grebe et al., 2000
	*AtCYP18-1*	NC_003070.9	ND	Heat	Sakuma et al., 2006
	*AtCYP18-3 (ROC1)*	NC_003075	–	Salt	He et al., 2004
	*AtCYP20-2*	NC_003076.8	PPIase activity	High irradiance	Romano et al., 2004a; Edvardsson et al., 2007
	*CYP38*	NM_111014.4	PPIase inactive	High light	Shapiguzov et al., 2006; Wang et al., 2015
*Brassica rapa*	*BrROC1 BrROC2*	NC_024800.1 KJ173687	ND	Cold, heat, dehydration, mannitol, salinity, light	Yan et al., 2018
*Cajanus cajan*	*CcCYP*	GU444041	PPIase activity	Salt, drought	Sekhar et al., 2010
*Capsicum annuum*	*CACYP1*	AF291180	ND	Salicylic acid, MeJA, ethylene and pathogen	Kong et al., 2001
*Digitalis lanata*	*DLCYP18.0/DLCYP18.1*	Y08320.1	PPIase activity	Abscisic acid, sorbitol	Küllertz et al., 1999
	*DLCYP*	Y08320	ND	PbN0_3_ and salt	Scholze et al., 1999
*Gossypium hirsutum*	*GhCYP1*	GQ292530.1	ND	Salt stress, biotic stress	Zhu et al., 2011
*Nicotiana tabacum*	*Cyclophilin-like protein*	EF495223.1	–	Induced by low nitrogen	Yang et al., 2013
*Oryza sativa*	*OsCyp2*	EF576508	PPIase activity	Salinity, high temperature, osmotic stress and oxidative stress	Kumari et al., 2009, 2015; Ruan et al., 2011
	*OsCYP18-2*	AK072675	PPIase activity	Drought	Lee et al., 2015b
	*OsCYP19-4*	NM_001052252	PPIase activity	Cold stress	Yoon et al., 2016
	*OsCYP20-2*	LOC_Os05g01270.1	PPIase activity	Osmotic stress	Kim et al., 2012
	*OsCYP21-4*	JC627182	PPIase inactive	Salt	Lee et al., 2015a
	*OsCYP25*	LOC_Os09g39780	PPIase inactive	Salt, heat, cold and drought	Trivedi et al., 2013a
*Solanum commersonii*	*ScCYP*	U92087	ND	Low temperature, abscisic acid and drought	Meza-Zepeda et al., 1998
*S. tuberosum*	*StCYP*	JX576267.1	ND	Heat, MeJA and abscisic acid	Godoy et al., 2000
*Thellungiella halophila*	*ThCYP1*	AY392408	ND	Salt	Chen et al., 2007
*Triticum aestivum*	*TaCYPA-1/TaCYP18-4*	JQ678695	PPIase activity	Heat stress	Kaur et al., 2016
	*TaCYP56-1*	TraesCS3A01G209000.1	ND	Heat stress	Singh et al., 2019
	*TaCYP64-4*	TraesCS4A01G045200.1	ND	Heat stress	Singh et al., 2019
*Vicia faba*	*pCYPB*	L32095	PPIase activity	Heat	Luan et al., 1994
*Zea mays*	*ZmCYP15*	Zm00001d050635	ND	Abiotic stress	Wang et al., 2017
**Animal**					
*Rattus rattus*	*CYPD*	NM_001004279.1	PPIase activity	Oxidative stress	Lin and Lechleiter, 2002
**Algae**					
*Cochlodinium polykrikoides*	*CpCYP*	ABX0001	–	Biocides, CuSO4 and NaOCl	Abassi et al., 2017
*Chlorella* sp.	*CsCYP1A*	KY207381	PPIase activity	NaHCO_3_, NaCl, and sorbitol stress	Liu et al., 2020
*Chlamydomonas reinhardtii*	*pCyP*	NW_001843852	–	Low carbon dioxide	Somanchi and Moroney, 1999
*Griffithsia japonica*	*GjCyp-1*	AF078071	Chaperonic activity	Heat stress	Cho et al., 2005; Cho and Kim, 2008
*Pyropia seriata*	*PsCYP1*	KU984106	ND	Salt and heat tolerance	Lee et al., 2017
*Porphyra haitanensis*	*PhCYP18*	JQ413239	ND	Salt stress and irradiance stress	Jia et al., 2013
*Prorocentrum minimum*	*PmCYP*	JF715159.1	–	Copper chloride and polychlorinated biphenyl	Ponmani et al., 2015
**Fungi**					
*Piriformospora indica*	*PiCYPA*	GQ214003	PPIase activity	Salt, cold, heat, cadmium chloride, cobalt chloride and hydrogen peroxide	Trivedi et al., 2013c
*Saccharomyces cerevisiae*	*CYP1, CYP2*	NC_001144.5 NC_001140	–	Heat	Sykes et al., 1993
	*CPR1*	KZV12392.1	–	Cadmium, copper, hydrogen peroxide, heat, SDS and oxidative stress	Kim et al., 2010

*The nomenclature used below is as given in literature. MeJA, methyl jasmonate; ND, not determined; SDS, sodium dodecyl sulfate.*

The ability to confer tolerance against a broad range of abiotic stress conditions was also observed for the rice cyclophilin OsCYP2 (Table 7), which is localized to cytosol and nucleus, and shares 62.79 % and 32.08 % identity with OsCYP19-4 and OsCYP21-4, respectively (Kumari et al., 2013, 2015). The *OsCYP2*-induced tolerance to stress in transgenic tobacco plants was attributed to the regulation of ion homeostasis due to an enhanced K^+^/Na^+^ ratio (Kumari et al., 2015). The drought tolerance in the OsCYP18-2 over-expressing transgenic *Arabidopsis*, on the contrary, was ascribed to reduced transpiration rate due to a decrease in stomatal aperture (Lee et al., 2015b). Though OsCYP18-2 was also shown to interact with the Ski interacting protein (OsSKIP) in rice (Lee et al., 2015b), the role of this interaction in stress tolerance is not understood. The abrogation of this interaction by engineering OsCYP18-2 and OsSKIP will provide further insights into its functional significance.

The plastidic cyclophilins have also been demonstrated to impart protection against stress. Ectopic expression of the thylakoid localized cyclophilins, OsCYP20-2 and AtCYP38, resulted in enhanced tolerance to various abiotic stresses in the transgenic *Arabidopsis* and tobacco plants (Kim et al., 2012; Wang et al., 2015; Ge Q. et al., 2020). While the OsCYP20-2-induced-tolerance was ascribed to higher chloroplast PPIase activity and maintenance of NADH dehydrogenase-like complex that protects the stroma against over-reduction under stress conditions, the AtCYP38-stimulated protection against high light intensity was due to inhibition of PsbO_2_ activity which is an important component of photosystem II (Wang et al., 2015). Recent studies have demonstrated the presence of two different variants of OsCYP20-2 in rice, and the two isoforms contribute to chilling stress tolerance through different mechanisms (Ge Q. et al., 2020). While the chloroplast-localized OsCYP2 contributes to scavenging of ROS by enhancing the activity of a superoxide dismutase, OsFSD2, the nuclear-localized isoform, generated following truncation of the chloroplast signal peptide, interacts with a DELLA protein, SLENDER RICE1, and stimulates its degradation to promote growth. These studies, hence, highlight the crucial role of OsCYP20-2 in integrating plant growth and abiotic stress response. As observed in transgenic tobacco plants that constitutively expressed *GjCYP-1*, a cyclophilin gene from red alga *Griffithsia japonica*, the PPIase-induced stress tolerance might also be associated with adverse effects on growth and yield (Cho and Kim, 2008), thereby, necessitating the use of stress-inducible promoters.

Though molecular processes underlying the cyclophilin-induced stress tolerance are not fully understood for the majority of the cyclophilins, prevention of protein aggregation, as reported for GjCYP-1, may be one of the protective mechanisms (Cho et al., 2005). The heat stress tolerance in *E. coli* that overexpressed a redox-regulated wheat cytosolic cyclophilin, TaCYPA-1, was however attributed to its PPIase activity (Kaur et al., 2016, 2017). Since the redox status of plants undergoes reversible changes under stress conditions (Jubany-Mari et al., 2010), application of a redox-sensing GFP (c-roGFP1) for real-time monitoring of cytosol redox status (Brossa et al., 2013) is needed to explore the role of TaCYPA-1 in the maintenance of redox homeostasis in the cell under stress conditions. Further, our studies also demonstrated that TaCYPA-1 and AtCYP19-3, that are 74 % identical, interact with calmodulin (CaM) in a Ca^2+^-dependent fashion (Popescu et al., 2007; Kaur et al., 2015). As Ca^2+^ is a transducer of stress signals (Snedden and Fromm, 2001; Virdi et al., 2015), cyclophilins may likely constitute an important component of Ca^2+^-CaM signaling pathway. Whether interaction with CaM is a property shared by all cyclophilins is still a matter of speculation and requires further investigations for elucidating the role of these proteins in CaM-mediated responses

The expression of cyclophilin genes is also regulated by CO_2_ and nitrogen. Transcript levels of a tobacco cyclophilin gene were reported to increase under low nitrogen conditions (Yang et al., 2013), but the physiological implication of this observation is yet to be ascertained. Due to the competitive nature of ribulose-1, 5-bisphosphate carboxylase oxygenase (Rubisco) catalyzed carboxylation and oxygenation reactions, the photosynthetic activity is low in plants and algae. Hence, under low CO_2,_ the CO_2_-concentrating mechanism (CCM) is induced in several algae such as *Chlamydomonas reinhardtii* (Moroney and Ynalvez, 2007). CCM leads to a high ratio of CO_2_ to O_2_ at the site of Rubisco and stimulates the carboxylation reaction under depleted CO_2_ conditions (Badger et al., 1980; Moroney and Mason, 1991). The establishment of CCM under low CO_2_ conditions in *C. reinhardtii* was reported to coincide with a transient increase in expression of a cyclophilin gene, indicating its likely role in this mechanism (Somanchi and Moroney, 1999). It was conjectured that this cyclophilin may be required for protecting the proteins against photodamage since CO_2_ is an electron receptor and a decrease in CO_2_ concentration at the same light imposes photooxidative stress. Similar roles cannot be ruled out for other cyclophilins, particularly the chloroplast-localized ones, and warrants further experimentation.

The cyclophilins from extremophiles such as *Piriformospora indica* and *Thellungiella halophila* also offer an attractive alternative to improve stress tolerance in crop plants (Table 7) (Chen et al., 2007; Trivedi et al., 2013b,c). PiCYPA cloned from the xerophytic fungus *P. indica*, despite lacking the canonical RRM, demonstrated interaction with RNA. It is likely that protection against stress in the *PiCYPA*-overexpressing transgenic *E. coli* and tobacco plants might be due to its role in the stabilization of RNA transcripts (Trivedi et al., 2013b). Induction of a 17.5 kDa cyclophilin *PmCYP* in dinoflagellate algae *Prorocentrum minimum* in response to different pollutants *viz*., copper and polychlorinated biphenyl (Ponmani et al., 2015) further suggests that the role of cyclophilins as stress proteins is conserved. The role of cyclophilins as universal stress proteins is also substantiated by studies on *Brucella*, an intracellular bacterial pathogen in humans and cows which causes the disease brucellosis (Young, 1995). Comparative proteomic analysis in *B. abortus* resulted in the identification of two cyclophilins (CYPA and CYPB) which were differentially expressed and implicated in bacterial intracellular adaptation (Roset et al., 2013). Studies employing Δ*cyp*AB mutants revealed that these genes were essential for virulence and tolerance to various abiotic stresses such as oxidative, acidic pH and detergents (Roset et al., 2013).

It is apparent that despite being distinct, protection against stress-induced damage is a property common to several cyclophilins (Table 7), suggesting an overlap of their roles. However, the precise mechanisms by which these proteins protect the cellular machinery against stress-induced damage are still elusive for the majority of these proteins. Although except for AtCYP38, all the cyclophilins implicated in stress tolerance are SD proteins, similar roles for the MD cyclophilins cannot be ruled out and should be the subject of future studies. Further investigations are therefore necessary to unravel the physiological implications of cyclophilins in plants that will enable their applications in crop improvement through biotechnological interventions or conventional breeding.

## Future Prospects

The characterization of cyclophilins in plants is revealing new insights into their physiological relevance. The presence of large gene families suggests that these cyclophilins might have overlapping yet distinct functions which are still speculative. As signified by analyses of available genomic data, the cyclophilin genes in plants display substantial variability in their structure, particularly in the context of the distribution of introns. Since introns play a role in regulating gene expression, rigorous studies are required to understand the implications of these differences in the regulation of cyclophilin genes. These studies are likely to provide insight into their physiological role. Despite the presence of conserved CLD, the presence of different domains such as TPR, WD, RRM, etc., in the MD cyclophilins indicate the acquisition of novel functions. However, the role of these domains in imparting specific functionalities to cyclophilins is still conjectural for the majority of these proteins. Therefore, it is imperative to carry out the targeted deletion of different motifs in MD cyclophilins of plants and analyze the effect thereof on various facets of growth and development. Despite high sequence similarity, variability in the structure of cyclophilins has been reported to result in dramatic changes in their biochemical properties. Given the diversity in plant cyclophilins, it is imperative to elucidate the structure of these proteins by using different biophysical approaches such as X-ray diffraction and nuclear magnetic resonance to identify their mechanism of action. Both PPIase active and inactive (AtCYP38) cyclophilins have been reported to play specific roles in plants, thus, rendering the role of PPIase activity in plants a matter of speculation. Hence, the expression of site-directed mutants that show graded PPIase activity might illustrate the precise function of this biochemical attribute in the plants. Since PPIase activity of several cyclophilins is regulated by different redox mechanisms, and several of these proteins are induced by stress that affects the redox status of the cell, investigations should also be undertaken to comprehend their role in the maintenance of redox-homeostasis. Though the cyclophilin-induced stress tolerance in plants has been attributed to their chaperonic functions, the detailed cellular mechanisms, with few exceptions, are yet to be deciphered. The chaperonic activities (holdase and foldase) of cyclophilins can be independent of PPIase function, due to which concerted efforts are required to characterize the different biochemical activities of plant cyclophilins and their implications in stress tolerance. The multifaceted nature of cyclophilins warrants multipronged approaches to delineate their mechanisms of action in plants.

## Conclusion

Compared with prokaryotes and animals, the cyclophilin gene families in plants have undergone dramatic expansion, implying functional diversification and their importance for different growth and developmental processes. Being sessile, the divergence of cyclophilins may enable the plants to respond and adapt to adverse environmental conditions since several of these genes are responsive to different abiotic and biotic stressors. It is evident that though the roles of majority of the cyclophilins in plants are obscure, these proteins by virtue of their PPIase and chaperonic activities are likely to regulate diverse aspects of growth and development. Furthermore, presence of additional functional domains such as WD, F-box, RRM, and Zn-knuckle might enable these proteins to facilitate assembly of multiprotein complexes and modulation of cellular processes through transcriptional, post-transcriptional, translational and post-translational regulation of gene expression, thereby, enabling them to play multifaceted roles in the cell. Studies carried out so far also reveal that the enzymatic activity of cyclophilins is regulated through diverse mechanisms that might be redox-dependent or independent, the physiological significance of which is still a matter of speculation. The implications of cyclophilins such as LeCYP, TaCYP20-2 and AtCYP18-3 in auxin, GA and brassinosteroid signaling further underline their functional versatility and indispensability for the plants. The studies carried out until now have though provided novel insights into the functional and regulatory aspects of plant cyclophilins, the physiological significance of the majority of these proteins is still a matter of conjecture. Therefore, concerted efforts are imperative to understand the importance of different cyclophilins in plants so that these genes can be used for the improvement of different traits in the crop plants.

## Author Contributions

HS: methodology, visualization, data curation, and writing-original draft preparation. KK: data curation, validation, visualization, and writing-original draft preparation. MS and GK: writing-original draft preparation and validation. PS: conceptualization, supervision, methodology, and reviewing and editing. All authors contributed to the article and approved the submitted version.

## Conflict of Interest

The authors declare that the research was conducted in the absence of any commercial or financial relationships that could be construed as a potential conflict of interest.
