# Supplementary figures and images for: Plant Cyclophilins: Multifaceted Proteins With Versatile Roles
Source: Front Plant Sci. 2020 Oct 22;11:585212. doi: 10.3389/fpls.2020.585212 (PMC7641896; doi:10.3389/fpls.2020.585212)

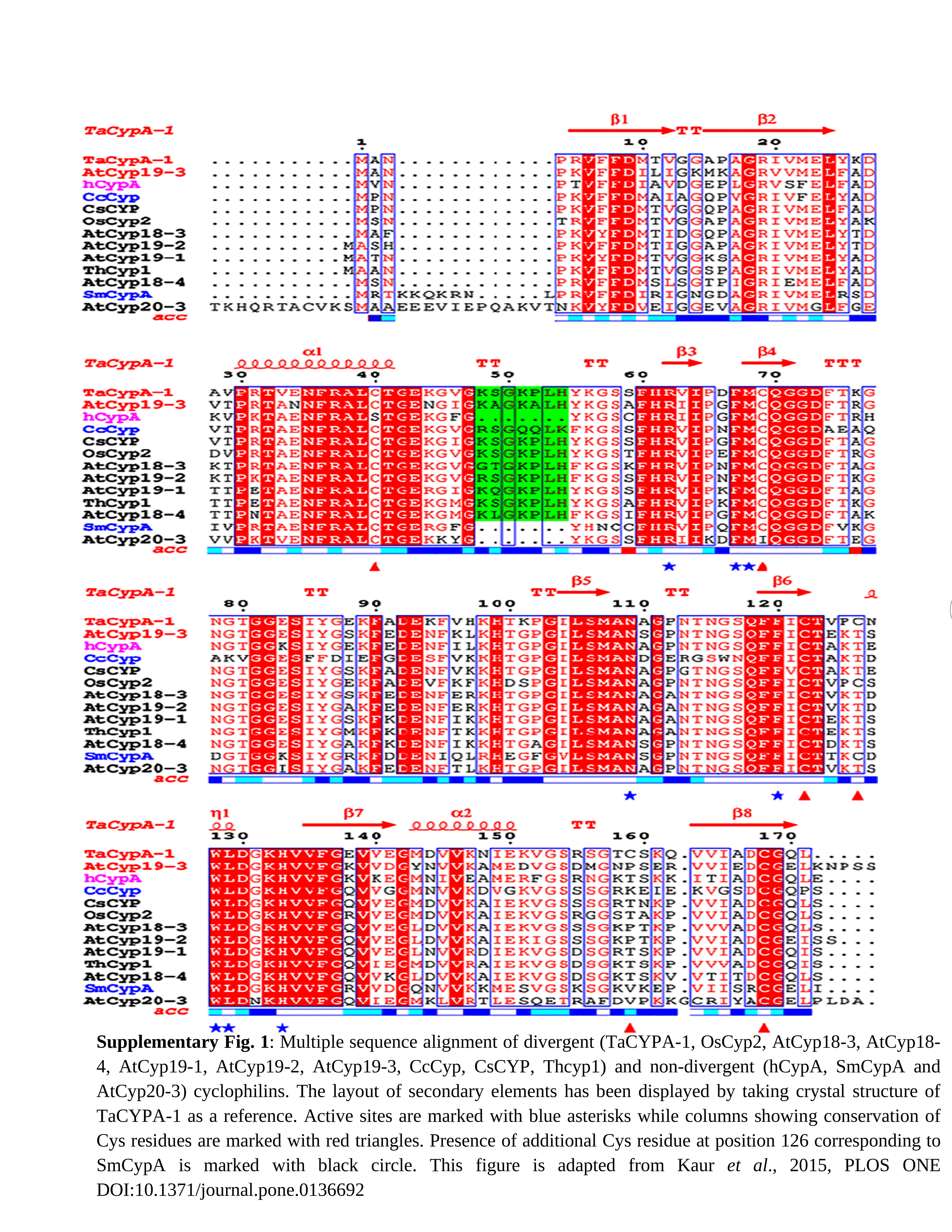

Supplement: Supplementary file 1 [file Image_1.TIF]
